# Job satisfaction has differential associations with delay discounting and risk-taking

**DOI:** 10.1038/s41598-023-27601-8

**Published:** 2023-01-14

**Authors:** Markus J. T. de Ruijter, Amelia D. Dahlén, Gull Rukh, Helgi B. Schiöth

**Affiliations:** 1 Department of Surgical Sciences, Uppsala University, Uppsala, Sweden; 2 Present Address: Department of Public Health and Caring Sciences, Geriatrics, Uppsala University, Uppsala, Sweden

**Keywords:** Psychology, Risk factors, Occupational health, Epidemiology

## Abstract

Low job satisfaction has been associated with both negative health and negative organizational outcomes. Knowledge on which factors influence job satisfaction remains limited. This study assesses the associations between job satisfaction and three personality traits related to cognitive- and inhibitory control: delay discounting, risk-taking and sensation seeking (DRS-traits). Delay discounting and sensation seeking were inferred using self-reported behavioral data and health measurements for 80,676 participants in the UK Biobank. Multiple linear regression analysis produced beta coefficients and confidence intervals for each DRS-trait and job satisfaction. Analyses were adjusted for age, socioeconomic status and sleep quality. A combination of the three DRS-traits (CDRS) was assessed as well. Delay discounting and risk-taking were associated with, respectively, lower and higher job satisfaction in both sexes. Sensation seeking had no significant association with job satisfaction for either sex. The combined score, CDRS, was only negatively associated with job satisfaction in females but not in males. We discuss that the negative association between delay discounting and job satisfaction may be due to career related delay discounting effects, but also highlight that low job satisfaction itself may also lead to increased delay discounting. Additionally, we discuss why increased risk-taking behavior may have a positive effect on job satisfaction.

## Introduction

Social and environmental factors have long been known to contribute to the incidence of many human diseases. Worldwide, many people spend a significant amount of their waking hours at the workplace and it is becoming increasingly clear that workplace associated pressure, strain and stress are important health factors. A comprehensive review by Faragher et al. revealed various health issues associated with low job satisfaction^1^. These include mental health issues such as burnout, depression and anxiety disorders, but also physical illness such as increased risk of cardiovascular disease. Even from an organizational point of view, high job satisfaction provides several benefits such as enhanced job performance and increased organizational citizenship behaviors^2^ (i.e., performing extra duties outside the formal job description). Factors that have been correlated to job satisfaction include organizational factors such as workload and empowerment^3^, but also include individual characteristics such as autonomy, education level^4^, and several of the “Big Five” personality traits^5,6^.

The Big Five personality traits consist of five superordinate bipolar traits that characterize an individual’s personality. It includes the traits: (1) neuroticism, (2) extraversion, (3) conscientiousness, (4) openness (to experience), and (5) agreeableness. A comprehensive review by Judge et al. shows that neuroticism is negatively associated with job satisfaction, while extraversion, agreeableness and conscientiousness are positively associated with it^5,6^. Interestingly, these Big Five traits are also associated with inhibitory control, and personality traits associated with inhibitory control: impulsivity (delay discounting in particular), risk-taking and sensation seeking (DRS-traits, further explained below)^7–11^. However, while many prior studies examined the association between the Big Five personality traits and job satisfaction, we found no comprehensive research that specifically examines the association between these DRS-traits and job satisfaction. As definitions of the traits differ in the literature, and tend to overlap, we give brief descriptions of the traits as they are used in this article.

*Impulsivity* has been defined as a “non-reflective stimulus-driven action when a later-rewarding goal-relevant response was also available”^7^. An important aspect of impulsivity relevant to this article, is *delay discounting*. Delay discounting refers to the “discounting” of rewards later in time, i.e. lowering their perceived value, with respect to rewards closer in time. Practically, this implies preferring a smaller-sooner reward as opposed to a larger-later reward, or with respect to negative outcomes, preferring a larger-later aversive outcome over a smaller-sooner one^12^. While delay discounting may present a failure to maximize reward, delay discounting in an evolutionary context can have benefits when it is uncertain if a larger-later reward can be obtained at all. For example, an animal quickly choosing a sub-optimal hiding place might be better off than searching longer for a more optimal hiding place, if it would not have survived long enough to find it^13^. So, while delay discounting may be beneficial when the future is uncertain, with the relatively certain future people have nowadays, delay discounting is typically associated with negative outcomes such as obesity and addiction^14,15^, but also lower salary^16^.

*Risk-taking* has been defined as “the action that discounts the probability of negative consequences relative to probability of positive consequences” (Nigg, 2017, p. 371). Thus, while delay discounting discounts rewards *temporally*, risk-taking discounts reward *probabilities*. Specifically, the probabilities of negative outcomes are discounted while probabilities for positive rewards are overestimated. Risk-taking behavior exists for several reasons. For example, to attain reward outcomes otherwise unattainable, in an attempt to gain an advantage over competitors, but also as a demonstration of ability^17^. In our current society, risk-taking has often been related to negative outcomes such as risky sexual behavior or gambling. However, recent studies have also begun to identify that certain risk-taking behavior may be viewed as positive by peers, an example of which can be seen in extreme sports^18^.

According to Marvin Zuckerman, *sensation seeking* is “a trait defined by the seeking of varied, novel, complex, and intense sensations and experiences, and the willingness to take physical, social, legal, and financial risks for the sake of such experience” (Zuckerman, 1994, p. 27). So, rather than relating to the concepts of discounting reward value or probability, sensation seeking primarily involves an individual's need for stimulation, or arousal^20^. From an evolutionary viewpoint, arousal is thought to act as a coupling mechanism between an organism's internally based needs system, with an externally based situational awareness system and forms the basis for consciousness^21^. Mechanically, arousal may evoke an emotional response which in turn motivates the body into motor action. An example would be a prey spotting an approaching predator, consequently raising its arousal levels, which lead to a fear emotion and a flight response. Sensation seeking may include risky behavior such as speeding, but also includes non-risky behavior such as a preference for arousing music and surreal or violent art forms^22^. Sensation seeking may also include social contexts such as peer-influenced alcohol consumption and drug use. With respect to vocation, sensation seekers prefer occupations involving novel, stimulating, and unconventional tasks with a greater amount of flexibility.

In today’s society, high delay discounting, risk-taking or sensation seeking are considered traits that affect an individual’s decision making process^7^. These traits may also be considered to result in a breakdown of that decision making process. That is, they may affect decisions in a way that an option with a suboptimal reward is selected. Behavior related to these traits may overlap and are often found to have a negative impact on health. This includes behavior such as over-eating^14,23^, more sedentary behavior and less physical activity^24–27^, risky acts of sex^28–31^, speeding^32,33^ and substance abuse^34–37^, which we further elaborate on in the methods section of this article.

There are various studies that show the influence of the DRS-traits on vocational choice and other work related aspects such as workplace safety and salary. At the same time, while psychological characteristics have been studied in conjunction with job satisfaction, associations with these specific DRS-traits have been severely understudied. In this study we investigated the associations between the DRS-traits and job satisfaction, regardless of profession, using the large scale UK Biobank Cohort (UKB)^38^. Delay discounting and sensation seeking traits were inferred using relevant variables available in the UKB. Together with the already available “Risk taking” variable we assessed the DRS associations with job satisfaction in a sample of mentally healthy employed individuals. Results of this study may highlight at-risk individuals and may be used to improve programs aimed at increasing job satisfaction within organizations, or career development of individuals.

## Results

### Demographics

After exclusion, 80,676 participants in the UKB were included in the present study (51.7% female, 48.3% male). Of the female participants, 8.3% reported being extremely happy in their jobs, 37.2% report being very happy, 46.4% being moderately happy, 6.1% being moderately unhappy, 1.4% very unhappy and 0.6% extremely unhappy. Of the male participants a similar trend is seen: 9.0% report being extremely happy, 36.5% being very happy, 43.7% moderately happy, 7.9% moderately unhappy, 2.0% very unhappy, 0.9% extremely unhappy. An overview of the demographics is shown in Table 1.

**Table 1:** Demographics of the study participants.

Characteristic	Female (n = 41,694)		Male (n = 38,982)	
Age (years)	53.9 (7.7)	40 | 70	54.8 (8.0)	40 | 70
Socioeconomic status (TDI)	− 1.4 (2.7)	− 6.3 | 9.1	− 1.4 (2.8)	− 6.3 | 9.3
Time in current job (years)	12.0 (9.6)	< 1 | 52	14.3 (11.6)	< 1 | 55
**Alcohol (%)**				
Never	3039 (7.3)		1940 (5.0)	
Twice or less per week	22,262 (53.4)		16,553 (42.5)	
At least three times per week	16,393 (39.3)		20,489 (52.6)	
**Smoking (%)**				
Never	26,078 (62.5)		21,176 (54.3)	
Previous	12,821 (30.8)		14,252 (36.6)	
Current	2795 (6.7)		3554 (9.1)	
Body mass index (kg/m^2^)	26.7 (5.0)	14.5 | 67.3	27.7 (4.1)	16.3 | 61.5
Vigorous exercise frequency (days/week)	1.8 (1.8)	0 | 7	2.2 (2.0)	0 | 7
**Driver faster than the speed limit (%)**				
Never/rarely	20,358 (48.8)		10,702 (27.5)	
Sometimes	16,651 (39.9)		17,819 (45.7)	
Often/always	4685 (11.2)		10,461 (26.8)	
Time spent watching TV (hours/day)	2.4 (1.4)	0 | 18	2.5 (1.4)	0 | 21
Number of sexual partners	5.6 (8.3)	1 | 500	11.5 (81.9)	1 | 12,000
**Falls (%)**				
None	33,196 (79.6)		33,517 (86.0)	
Once	6171 (14.8)		3837 (9.8)	
More than once	2327 (5.6)		1628 (4.2)	
Self reported risk-taker (yes) (%)	9596 (23.0)		14,910 (38.2)	
**Job satisfaction (%)**				
Extremely happy	3460 (8.3)		3505 (9.0)	
Very happy	15,517 (37.2)		14,215 (36.5)	
Moderately happy	19,334 (46.4)		17,028 (43.7)	
Moderately unhappy	2544 (6.1)		3095 (7.9)	
Very unhappy	580 (1.4)		789 (2.0)	
Extremely unhappy	259 (0.6)		350 (0.9)	

Values are represented as (1) mean (standard) min | max, or (2) absolute number (percentage).

After scaling the DRS-trait and CDRS scores into values between 0 and 1 (0 being the *lowest* value and 1 the *highest* value), we could compare the relative scores between each other and between both sexes. In females, we found a mean relative delay discounting score of 0.25 (± 0.22), a mean relative risk-taking score of 0.23 (± 0.42), a mean relative sensation seeking score of 0.20 (± 0.21), and a mean relative CDRS score of 0.25 (± 0.16). In males, we found a mean relative delay discounting score of 0.24 (± 0.21), a mean relative risk-taking score of 0.38 (± 0.49), a mean relative sensation seeking score of 0.31 (± 0.24), a mean relative CDRS score of 0.32 (± 0.18). A graphical overview of the relative scores is shown in Supplementary Fig. S1.

### DRS-traits and CDRS associations with job satisfaction

**Figure 1:**
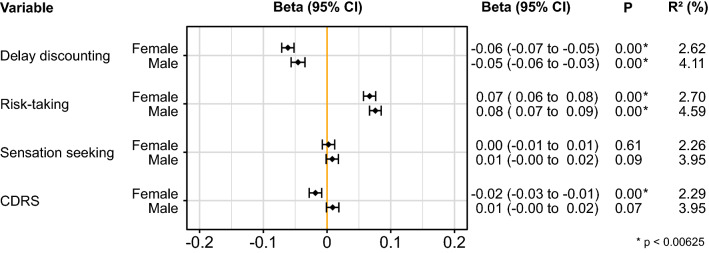
Beta estimates and confidence intervals (CI) of the associations between the DRS-traits and CDRS, with job satisfaction. The variables are standardized, thus representing the change in job satisfaction associated with each standard deviation increase of the respective variable. *CDRS* combined delay discounting, risk-taking and sensation seeking.

Standardized versions of the DRS-traits, CDRS and job satisfaction were used during multiple linear regression. Thus, these scores show the amount of change in job satisfaction that is associated with each standard deviation increase of the respective variable. An overview of the associations is shown in Fig. 1.

The model for delay discounting revealed a negative association between delay discounting and job satisfaction in both females [β: − 0.061; (CI − 0.071, − 0.052); (P = 2.29 × 10^–35^)] and males [β: − 0.045; (CI − 0.056, − 0.034); (P = 2.66 × 10^–16^)]. The risk-taking model showed a positive association between risk-taking and job satisfaction in both females [β: 0.067; (CI 0.057, 0.077); (P = 1.09 × 10^–42^)] and males [β: 0.076; (CI 0.067, 0.085); (P = 1.51 × 10^–59^)]. The model for sensation seeking revealed no significant association between sensation seeking and job satisfaction in either females [β: 0.003; (CI: − 0.007, 0.012); (P = 0.61)] or males [β: 0.008; (CI − 0.001, 0.018); (P = 0.09)]. Finally, the model for CDRS showed that CDRS was associated with lower job satisfaction in females [β: − 0.018; (CI − 0.028, − 0.008); (P = 3.18 × 10^–4^)], but no significant association was found for males [β: 0.009; (CI − 0.001, 0.019); (P = 0.07)]. A full model, including all covariates, sex, and the DRS-traits, is shown in Supplementary Fig. S2. An overview of the associations between the individual covariates, age, TDI, and sleep, with job satisfaction is shown in Supplementary Fig. S3. The raw data of the DRS-traits versus job satisfaction is shown in Supplementary Fig. S4.

## Discussion

To our knowledge, this is the first large-scale study to examine associations between delay discounting, risk-taking and sensation seeking, with job satisfaction, regardless of profession. We found that delay discounting and job satisfaction share a negative association while risk-taking and job satisfaction have a positive association. Sensation seeking was not associated with a difference in job satisfaction, while our combined score for the three traits, CDRS, showed a relatively small negative association with job satisfaction in females only.

Characteristics of delay discounting include prioritizing an immediate reward over a potential greater reward in the future, but also includes poor future planning^7^. Although we cannot infer causation in this study, the negative association between delay discounting and job satisfaction may be the result of both a cause and a consequence of low job satisfaction. Two studies found that delay discounting occurs in relation to job choice, for example when considering salary or preferred tasks^39,40^. Additionally, better coping with delay of gratification is shown to have a positive effect on job performance^41^. Another study highlights that “delay discounting individuals” are less likely to find a satisfactory career path^42^. All these factors might eventually lead to delay discounting individuals having lower job satisfaction. Conversely, low job satisfaction has been shown to increase behavior associated with delay discounting. A study by Herman et al. shows that negative emotions could lead to increased temporal delay discounting. They note that people in a positive mood are more likely to make long term commitments such as diets and regular exercise. Particularly, they highlight that the negative emotional state may lead to a preference for immediate gratification, such as the need for comfort food, watching television and foregoing exercise^43^. As poor job satisfaction likely leads to negative emotions, a causal relationship between poor job satisfaction and increased delay discounting behavior also seems plausible. Several other studies highlight the positive association between stress and subgroups of impulsivity, further supporting the idea that poor job satisfaction may eventually lead to delay discounting behavior^44,45^.

Risk-taking behavior entails the overestimation of the probability of positive outcomes while discounting probabilities of negative outcomes. Our study reveals a positive correlation between risk-taking and job satisfaction. Unfortunately, there exists little direct research about the association between risk-taking behavior and job satisfaction. However, several relevant studies found a correlation between risk-taking and pro-social outcomes^46,47^. This includes so-called *“positive risk-taking”*, which occurs when risk-taking behavior is positively received by peers or even glorified^18,48^. It seems plausible to believe greater social acceptance may lead to increased job satisfaction. Risk-taking has also been linked to a greater sense of agency^49,50^. A higher sense of agency increases the possibility to influence one's own work, which in turn may lead to a higher sense of involvement, an important factor of job satisfaction^3,51^. In relation to this, risk-takers seem to be more likely to be in positions where they have greater autonomy or control over their work. This includes being self-employed or having leadership roles^52,53^. Finally, we may speculate that, compared to non-risk-takers, risk-takers are more likely to take chances in changing aspects of their life or work when they are unsatisfactory, for example by changing their tasks or jobs.

In comparison to other studies, it is interesting to see that differential associations for delay discounting or impulsivity, and risk-taking are not uncommon. Particularly, Panwar et al. identified differential associations of impulsivity and risk-taking on brain activations underlying working memory^54^. Herman et al. found differential associations of these traits with interoception and mood states, linking risk-taking to positive affect, and temporal impulsivity to negative affect^43^. When zooming in on specific professions, it is worth mentioning that results may differ. We found two studies that found a negative association between risk-taking and job satisfaction. The first study examined a small Dutch cohort of sex workers^55^. The second study examined employees in a Norwegian transport company during a time of organizational change^56^.

The strengths of this study include the use of the large UK Biobank cohort. The availability of official medical records allowed us to reliably exclude participants with mental or behavioral disorders, enabling us to study the associations between the DRS-traits and job satisfaction in otherwise mentally healthy individuals. Our fairly strict exclusion criteria meant that every participant included in the study had complete measurement data. This allowed us to reliably infer the delay discounting and sensation seeking proxy variables. Moreover, the large sample size made it possible to adjust for confounding factors in our models and conduct sex-specific analysis, while retaining high statistical power. Finally, to our knowledge this is the first study to examine the associations between all three DRS-traits and job satisfaction in such a large cohort, regardless of profession.

The use of proxies to infer delay discounting and sensation seeking could be considered a limitation of this study. As both of these proxies were defined using just four behavioral aspects of the participants, it is possible that not all aspects of delay discounting and sensation seeking are included in our traits. Risk-taking was defined using the self-reported (“Yes” or “No”) risk-taking assessment. This also poses a limitation in terms of accuracy (how much of a risk-taker someone is), and definition (how did participants interpret risk-taking?). We once more want to mention that the traits, particularly risk-taking and sensation seeking, tend to overlap, as is evident from two commonly used assessment forms^57,58^. This should be considered when comparing our results to other studies. Finally, all data contained in the UKB is derived from UK based individuals, thus extrapolating these findings to other nations or cultures should be done with care.

To conclude, data contained in the UK Biobank cohort allowed us to develop proxy variables for delay discounting and sensation seeking traits in participants. Together with the existing “Risk taking” variable we were able to examine associations between these DRS-traits, and job satisfaction. Analysis revealed a negative association between delay discounting and job satisfaction, but a positive association between risk-taking and job satisfaction. Differences between females and males could only be found when comparing the combined delay discounting, risk-taking and sensation seeking score, where a negative correlation was identified for females, but not for males. Ultimately, we hope that these results will be used in a collective effort to understand the mechanics behind job satisfaction, and how job satisfaction may be increased. Examples of this may include promoting more autonomy, flexibility or benefits for employees. Results may differ across different occupations. We believe that further job-specific investigations into job satisfaction will be highly beneficial in improving the wellbeing and careers of individuals, as well as the overall satisfaction within organizations.

## Methods

### Study population

**Figure 2:**
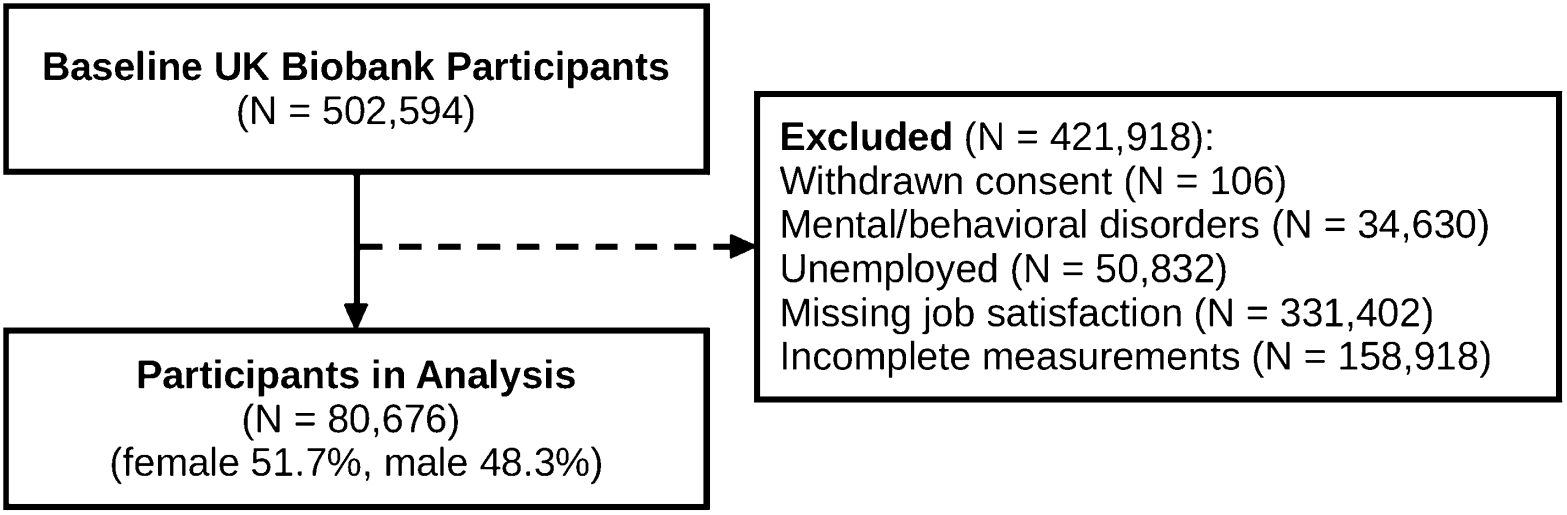
Overview of Study Sample.

The UKB is a population-based prospective cohort study with 502,594 participants (aged 37–73 years) that were recruited between 2006 and 2010 from 22 assessment centers across the United Kingdom (UK)^38^. The present study was carried out according to the Declaration of Helsinki. The UKB study was approved by the North West Multi-Centre Research and all participants provided written informed consent to participate in the UKB^38^. Ethical approval for this study was granted by the North West Multi-Centre Research Ethics Committee (11/NW/0382), the Regional Ethics Committee of Uppsala, Sweden (Dnr 2017/198). Details regarding the UKB have been described elsewhere^59^. Participants who had withdrawn consent (N = 106), had a history of mental or behavioral disorders (ICD 10 codes F00-F99 or ICD 9 codes 290–319. N = 34,630), or were unemployed (N = 50,832), were excluded from the analysis. As the registration of job satisfaction started after the initiation of data collection, not all participants had a job satisfaction score. Participants without job satisfaction scores were excluded from this analysis (N = 331,402). Finally, participants that responded to questions in ways that made it difficult to infer the DRS-traits, were considered to have incomplete measurements and were also excluded (N = 158,918). Examples of incomplete measurements include responses such as “Don’t know”, or “Prefer not to answer”. The final data set contained 80,676 participants. An overview of the study sample is shown in Fig. 2.

### Study measures

#### UKB variables related to the DRS-traits

In order to assess delay discounting, risk-taking and sensation seeking traits in participants, relevant variables were sought out in the UKB. Risk-taking could be directly inferred from the UKB variable “Risk taking”. This variable was part of the questionnaire, where participants were asked “Would you describe yourself as someone who takes risks?”. Possible answers included: “Yes”, “No”, “Don’t know”, or “Prefer not to answer”. No variables were present in the UKB from which delay discounting or sensation seeking could be inferred directly. Thus, these two traits were inferred by proxy, using other relevant data contained in the UKB.

Eight UKB variables were identified that have an association with any, or several, of the DRS-traits, or with aspects of cognitive- and inhibitory control. These included: “Alcohol intake frequency”, “Smoking status”, “Body mass index (BMI)”, “Lifetime number of sexual partners”, “Drive faster than motorway speed limit”, “Time spent watching TV”, “Falls in the last year”, and “Number of days/week of vigorous physical activity 10 + minutes”. In all cases, these variables were dichotomized and given a value of 1 if the answer selected by the participant was deemed delay discounting, risk-taking or sensation seeking, and a value of 0 if this was not the case. Paragraphs below elaborate on the variables used and how they were dichotomized. An overview of the variables used and the dichotomization criteria is shown in Table 2.

**Table 2:** Overview of study measures.

UKB variable	Unit/answer options^1^	Dichotomization^2^	DRS-trait
Time spent watching television (TV)	Hours/week	≥ 4	Delay discounting
Body mass index (BMI)	kg/m^2^	≥ 30	
Days *without* > 10 min of vigorous physical activity ^3^	Days/week	≥ 6	
Falls in the last year	0, 1 or > 1	> 1	
Risk taking	Yes/no	Yes	Risk-taking
Smoking status	Never, previous, current	Current	Sensation seeking
Alcohol intake frequency	Days/week	≥ 3	
Lifetime number of sexual partners	Number	≥ 7	
Drive faster than motorway speed limit	Never/rarely, sometimes, often, most of the time	Often, most of the time	

This table shows an overview of the UKB variables used to determine the DRS-traits. The CDRS variable consists of the sum of the dichotomized versions of all these variables.

^1^“Do not know” and “Prefer not to answer”, answer options were excluded from the analysis.

^2^Dichotomization, indicating delay discounting, risk-taking or sensation seeking (DRS) behavior, was performed using these criteria.

^3^The inverse of the actual UKB variable “Number of days/week of vigorous physical activity 10 + min”.

##### Alcohol consumption

The association between alcohol consumption and inhibitory control has been studied on several occasions. A weak or immature inhibitory function has been found to be related to higher alcohol consumption. However, alcohol consumption itself may also lead to lower impulse control. This suggests that the association between alcohol consumption and inhibitory control may be bidirectional^34,35^. Additionally, alcohol consumption has been associated with increased sensation seeking, where part of this association might be mediated by unstructured socializing with peers^60^. Furthermore, both the Domain-Specific Risk-Taking (DOSPERT) scale and Zuckerman’s Sensation Seeking Scale (SSS) include items involving alcohol use^57,58^. A dichotomized variable was created based on the UKB variable “Alcohol intake frequency (times per week)”. This variable was set to 1 if the intake frequency was 3 or more times per week, and 0 if it was less than 3 times per week.

##### Smoking

Balevich et al. revealed distinct levels of sensation seeking between individuals with different smoking habits^37^. “Never-smokers” had the lowest level of sensation seeking, while “smokers” had the highest. Lydon-Staley and Geier found both sensation seeking and inhibitory control to be associated with smoking habits^36^. According to their study, sensation seeking had the strongest association in adolescents, while inhibitory control had the strongest association in those in their mid 20’s and early 30’s. In addition, Evans-Polce et al. found sex-specific differences between the strength of the association between sensation seeking and smoking^61^. However, for both sexes, sensation seeking and smoking were consistently positively correlated throughout early adulthood. A dichotomized variable was derived based on the UKB variable “Smoking status”. The variable contained one of three answers: “Never”, “Previous”, and “Current”. The dichotomized variable was set to 1 if the participant chose “Current”, and 0 if “Never” or “Previous” were chosen.

##### Obesity

In systematic reviews by Schag et al., and Giel et al., over 70 publications related to impulsivity and obesity were compared^14,23^. The general conclusion was that one aspect of impulsivity, reward sensitivity, was increased in obese individuals when compared to normal weight controls. A dichotomized variable was created based on the UKB variable “Body mass index (BMI)”. This variable was set to 1 if a BMI of 30 or higher was reported. Individuals with a BMI of 30 or higher fall into the “obese” category.

##### Sexual activity

Several studies have found associations between the DRS-traits and risky sexual behavior^28–31^. Risky sexual behavior includes behavior such as unprotected sex, sex while intoxicated, and sex with multiple partners. Furthermore, DRS-traits had been found to have a positive correlation with the lifetime number of sexual partners^62^, and negative correlation with the age of sexual debut^63^. Items involving risky sex and sex with multiple partners are also included in the DOSPERT and SSS assessment forms^57,58^. A dichotomized variable was derived from the UKB variable “Lifetime number of sexual partners”. This variable was set to 1 if the number of sexual partners participants registered was in the upper tertile (7 partners or more).

##### Speeding

Several studies found associations between driving habits and DRS-traits. Drivers with higher levels of anger, sensation seeking, urgency, and with a lack of premeditation and perseverance are more likely to report higher levels of a broad range of risky driving acts^32^. Additionally, low inhibitory control was positively associated with speeding habits in a simulated driving experiment^33^. Two other studies compared people who were fined for speeding, to control groups. The first study found that those that speeded were more likely to score high on both the “Excitement Seeking” and “Fast Decision-Making” facets of the Adaptive and Maladaptive Impulsivity scale (AMIS), as well as the Overestimated Driving facet of the Driver Skill Inventory (DSI)^64^. The second study found that those that speeded scored lower on a Go/No-go test, which measures inhibitory control^65^. A dichotomized variable was created based on the UKB variable “Drive faster than motorway speed limit”. Relevant answer options for this variable included: “Never/rarely”, “Sometimes”, “Often”, “Most of the time”. The dichotomized variable was set to 1 if participants selected “Often” or “Most of the time”. Participants that did not drive on the motorway were excluded from the study.

##### Screen time

DRS-traits have also been associated with time spent watching TV, computer screens and smartphones. One study shows a positive association between time spent watching TV or being on the computer, and DRS-traits. Interestingly, they did not find this association when assessing video games specifically^24^. A study on children reveals that children with various forms of impulsivity are less likely to adhere to limitations of screen time^66^. Another study reveals an association between urgency and binge-watching^67^. Additionally, smartphone usage has been associated with higher delay discounting^68^. A dichotomized variable was created from the UKB variable “Time spent watching television”, measured in hours per week. This variable was set to 1 if participants registered an answer in the upper tertile (4 h or more per day).

##### Injury

Associations between DRS-traits and injury are inconsistent. The risk of injury has different associations between different facets of sensation seeking. Two studies assessing injuries in skiers show that sensation seeking, and thrill and adventure seeking were negatively associated with risk of injury^69,70^. However, another study regarding fall-risk in seniors found that high risk-taking individuals were more likely to engage in activity with a higher likelihood of falling^71^. Subsequently, they record an increased number of falls over the next year in high risk-taking participants. A study involving Canadian school children highlights that risk of injury might be more related to lack of risk perception as well as an overestimation of ability^72^. Despite contradictory evidence, we decided to create a dichotomized variable based on the UKB variable “Falls in the last year”. The dichotomized variable was set to 1 if participants registered more than one fall in the past year.

##### Exercise

Several studies have found relationships between physical activity and executive function^25–27^. Executive function is the psychological construct used to suppress impulsive or risky behavior. Interestingly, while higher executive function is associated with more physical activity, an increase in physical activity seems to increase executive function^73–75^. This thus suggests a bidirectional relationship. A dichotomized variable was created based on the UKB variable “Number of days/week of vigorous physical activity 10 + minutes”. However, as we are interested in delay discounting, risk-taking and sensation seeking, we thus seek the opposite of executive function. We therefore compute the inverse of this variable that we have called: “Number of days/week *without* vigorous physical activity 10 + minutes”. The dichotomized variable was set to 1 if participants registered a value of 6 days or more per week.

#### Delay discounting and sensation seeking proxies

Risk-taking had already been defined in the UKB. However, delay discounting and sensation seeking needed to be inferred from the DRS-relevant variables listed above. An intermediate analysis was performed on the DRS-relevant variables in order to determine if clusters could be identified computationally. The eight identified DRS-relevant variables were processed using a hierarchical clustering algorithm (Unweighted Pair Group Method with Arithmetic means^76^), the results of which are available in Supplementary Fig. S5. This resulted in a dendrogram with two main clusters. The first cluster contained the variables “Time spent watching television”, “BMI”, “Number of days/week *without* vigorous physical activity 10 + minutes” and “Falls in the last year”. Because most of these variables were related to the need for immediate gratification, we used this cluster as a representative of delay discounting. The second cluster included “Smoking status”, “Alcohol intake frequency", “Lifetime number of sexual partners” and “Drive faster than motorway speed limit”. As these variables seemed to be related to the desire for stimulation, particularly with respect to social behavior, we used the variable in this cluster to represent sensation seeking. Delay discounting and sensation seeking were computed by taking the sum of the dichotomized variables contained in their respective clusters.

We, thus, defined the individual DRS-traits: delay discounting and sensation seeking, each consisting of 4 variables, with a score ranging from 0 (*lowest*) to 4 (*highest*); and risk-taking, consisting of 1 variable, with a score of 0 (*lowest*) or 1 (*highest*). An overview of the UKB variables and to which trait they belong is shown in Table 2. In addition, correlations between the DRS-traits themselves are illustrated in Supplementary Fig. S6. An overview of the distribution of the scores between females and males is shown in Supplementary Fig. S1.

#### Combined DRS (CDRS) score

The different DRS-traits are found to have some overlapping behavior. An example of this is impulsive risk-taking where an individual engages in unplanned risky behavior without stopping to think, or the notion that risk-taking and sensation seeking correlate to some degree^7,57,58^. Because of this, we were interested in knowing if a combined delay discounting, risk-taking and sensation seeking score would provide further insight into the association between these traits and job satisfaction. Therefore, a combined delay discounting, risk-taking and sensation seeking (CDRS) score was also derived. This CDRS score was calculated by taking the sum of the eight dichotomized DRS-related variables as well as the “Risk taking” variable. This resulted in a new variable ranging from 0 (*lowest*) to 9 (*highest*). The distribution of the score among the participants is shown in Supplementary Fig. S1.

#### Study endpoint—job satisfaction

Our study endpoint, “job satisfaction”, was measured using a self-report item contained in the questionnaire. A multiple choice touchscreen question "In general how satisfied are you with the WORK that you do?" was presented to the participants. Possible answers included: “Extremely happy”, “Very happy”, ”Moderately happy”, ”Moderately unhappy”, ”Very unhappy”, ”Extremely unhappy”, ”I am not employed”, ”Do not know”, and ”Prefer not to answer”. Participants that answered “I am not employed”, “Do not know” or “Prefer not to answer” were excluded from the analysis. The remaining answers were given a numerical score from 0 to 5, where 0 represents the lowest job satisfaction (*Extremely unhappy*), and 5 the highest (*Extremely happy*).

### Study covariates

The covariates that were taken into account included age, socioeconomic status and sleep quality. UKB variable “Age at recruitment” was used to assess the participants age. For socioeconomic status, the UKB variable “Townsend deprivation index at recruitment” (TDI) was utilized. TDI represents the level of material deprivation and is computed based on the level of employment, non-car ownership, non-home ownership and household overcrowding in areas within the UK^77^. Sleep quality was based on the UKB variable “Sleep duration”, which was included in the self-report questionnaire with the question “About how many hours sleep do you get in every 24 h? (please include naps)”.

As several reports highlight the differences in propensity to engage in delay discounting, risk-taking or sensation seeking behavior between females and males^78,79^, we decided to stratify the analysis based on sex. The UKB variable “Sex” was used to determine the sex of the participant. This variable contained a mixture of the sex that the National Health Service had recorded for the participant, as well as self-reported sex.

### Statistical analysis

Descriptive statistics for continuous variables are presented as a mean (± standard deviation (*SD*) of the mean) and for categorical variables as number (percentage (%)). Associations between the DRS-traits and CDRS, and job satisfaction were analyzed using multiple linear regression. The output of the multiple linear regression includes beta estimates (β) and 95% confidence intervals (CI). Prior to performing the multiple linear regression, variables were standardized so that their means were 0 and *SD*s were 1. Thus, β represents the amount of change in job satisfaction that is associated with each per-standard-deviation increase of each respective trait. Separate models were created for each DRS-trait and CDRS, resulting in 4 models for each sex. All models included the three covariates age, socioeconomic status, and sleep quality, to correct for potential confounding. A full model, combining all covariates, sex, and the DRS-traits, is included as Supplementary Fig. S2. All statistical analyses were performed in R version 3.6.3^80^. Figures were generated in R using ggplot2^81^ and heatmaply^82^. To account for multiple testing for four variables (delay discounting, risk-taking, sensation seeking and CDRS) and two sexes, Bonferroni correction was applied and a p < 0.00625 (i.e. 0.05/8) was considered significant in all analyses.

### Ethics

The present study was carried out according to the Declaration of Helsinki. The UKB study was approved by the North West Multi-Centre Research and all participants provided written informed consent to participate in the UKB^38^. Ethical approval for this study was granted by the North West Multi-Centre Research Ethics Committee (11/NW/0382), the Regional Ethics Committee of Uppsala, Sweden (Dnr 2017/198).

## Supplementary Information

Supplementary Information.

## Data Availability

The data used in this study was obtained from the UK Biobank Cohort. Access to the UK Biobank data source can be obtained after successful registration and application process. Details can be found on the UK Biobank website: https://www.ukbiobank.ac.uk/
