# Supplementary material for: Job satisfaction has differential associations with delay discounting and risk-taking
Source: Sci Rep. 2023 Jan 14;13:754. doi: 10.1038/s41598-023-27601-8 (PMC9840618; doi:10.1038/s41598-023-27601-8)
Supplement: Supplementary file 1 — Supplementary Information. [file 41598_2023_27601_MOESM1_ESM.pdf]

Supplementary Data

Supplementary Figure S1. Distributions of the relative trait scores stratified by sex

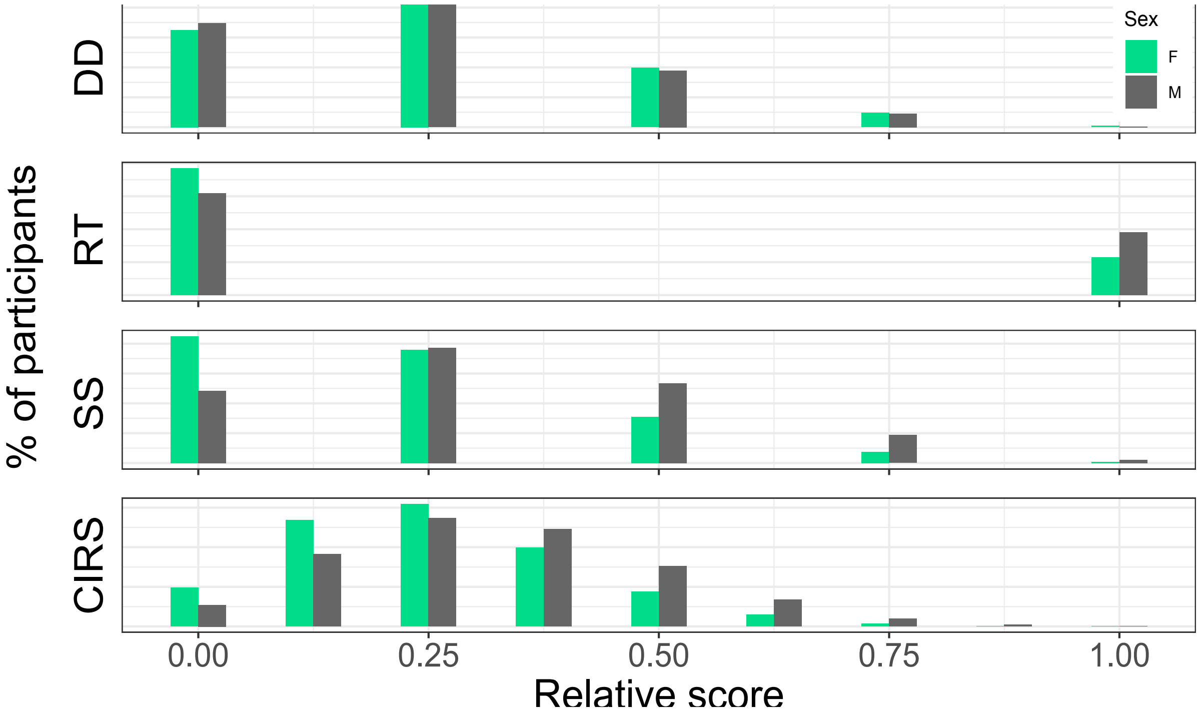

Histograms showing the trait scores scaled between 0 (lowest) and 1 (highest). F: female; M: male; DD: delay discounting; RT: risk-taking; SS: sensation seeking; CDRS: combined delay discounting, risk-taking and sensation seeking score.

Supplementary Figure S2. Forest plot of the full model

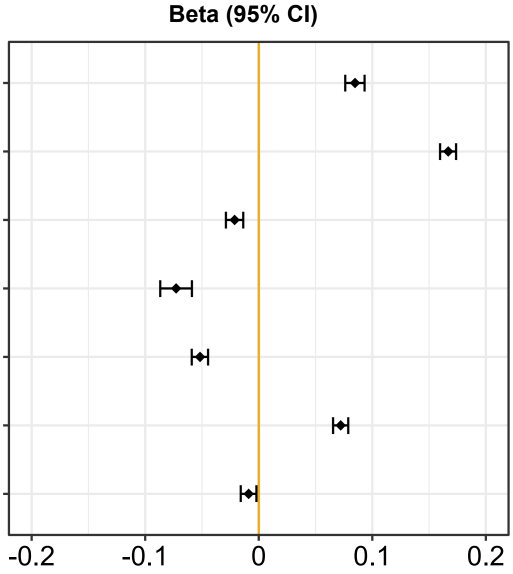

Variable	Beta (95% CI)	P
Sleep	0.08 (0.08 to 0.09)	0.00
Age	0.17 (0.16 to 0.17)	0.00
TDI	-0.02 (-0.03 to -0.01)	0.00
Sex (m)	-0.07 (-0.09 to -0.06)	0.00
Delay discounting	-0.05 (-0.06 to -0.04)	0.00
Risk-taking	0.07 (0.07 to 0.08)	0.00
Sensation seeking	-0.01 (-0.02 to -0.00)	0.01

This forest plot shows the results of the full model, which included all three covariates, sex, and all three DRS-traits. Beta estimates were obtained using multiple linear regression. TDI: Townsend deprivation index. R^2 = 3.9%

Supplementary Figure S3. Forest plot showing associations between the study covariates and job satisfaction

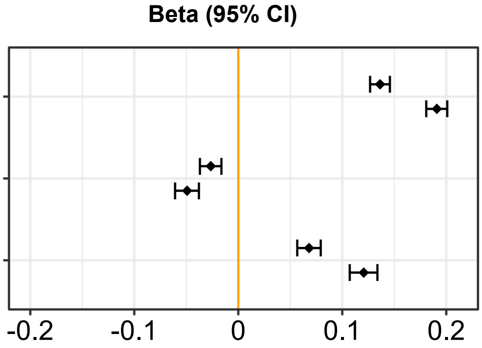

Variable		Beta (95% CI)	P	R^2 (%)
Age	Female	0.14 (0.13 to 0.15)	0.00	1.85
	Male	0.19 (0.18 to 0.20)	0.00	3.39
TDI	Female	-0.03 (-0.04 to -0.02)	0.00	0.06
	Male	-0.05 (-0.06 to -0.04)	0.00	0.18
Sleep	Female	0.07 (0.06 to 0.08)	0.00	0.34
	Male	0.12 (0.11 to 0.13)	0.00	0.81

Beta estimates were obtained using linear regression between the individual covariates and job satisfaction. TDI: Townsend deprivation index.

Supplementary Figure S4. Overview of the raw data of the IRS-traits and CIRS versus job satisfaction

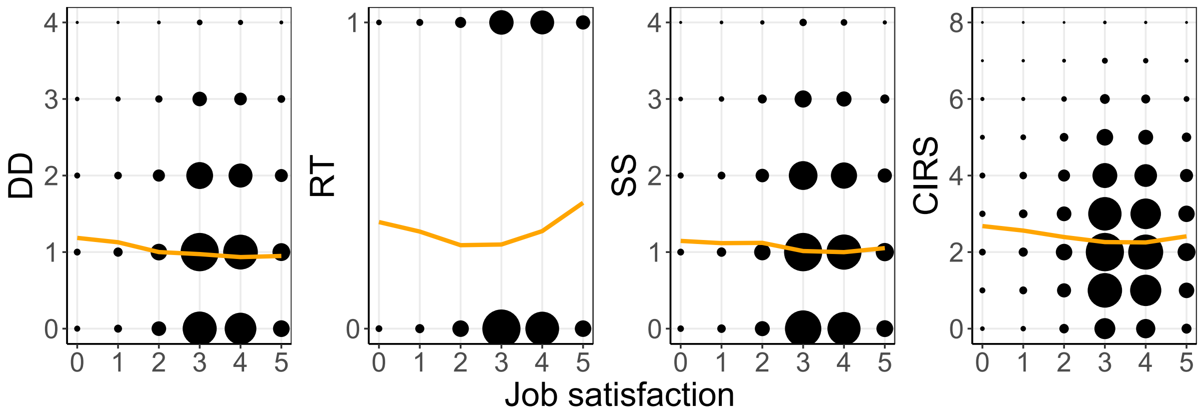

The point size represents the number of participants in that location. Notably, most participants registered a job satisfaction score of 3 or 4 (respectively, *moderately* and *very happy*). The line represents the mean score of the trait for each level of job satisfaction. This is the combined data for females and males. DD: delay discounting; RT: risk-taking; SS: sensation seeking; CDRS: combined delay discounting, risk-taking and sensation seeking score.

Supplementary Figure S5. Correlation plot of the proxy variables

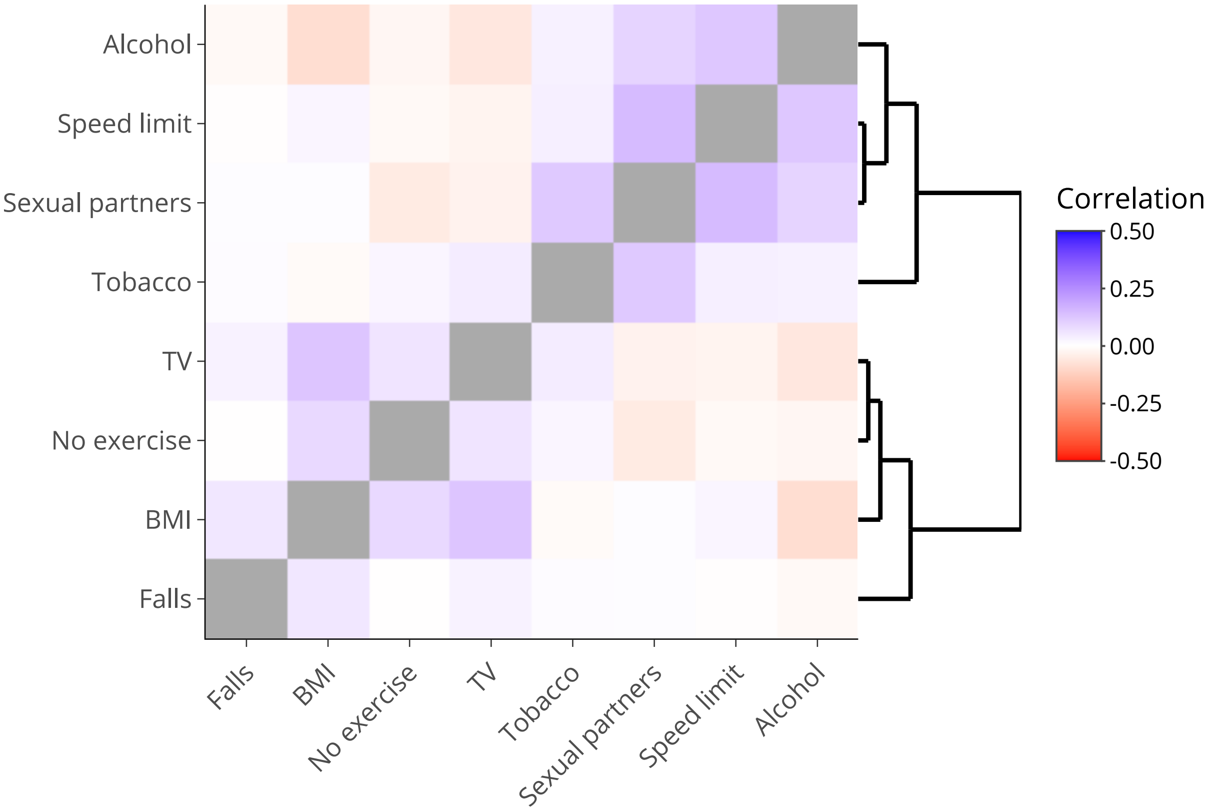

This figure shows the correlation between the DRS-relevant variables. The dendrogram was computed using the Unweighted Pair Group Method with Arithmetic means algorithm. The top branch was used to represent sensation seeking, while the bottom branch was used to represent delay discounting.

Supplementary Figure S6. Correlation plot of the DRS-traits

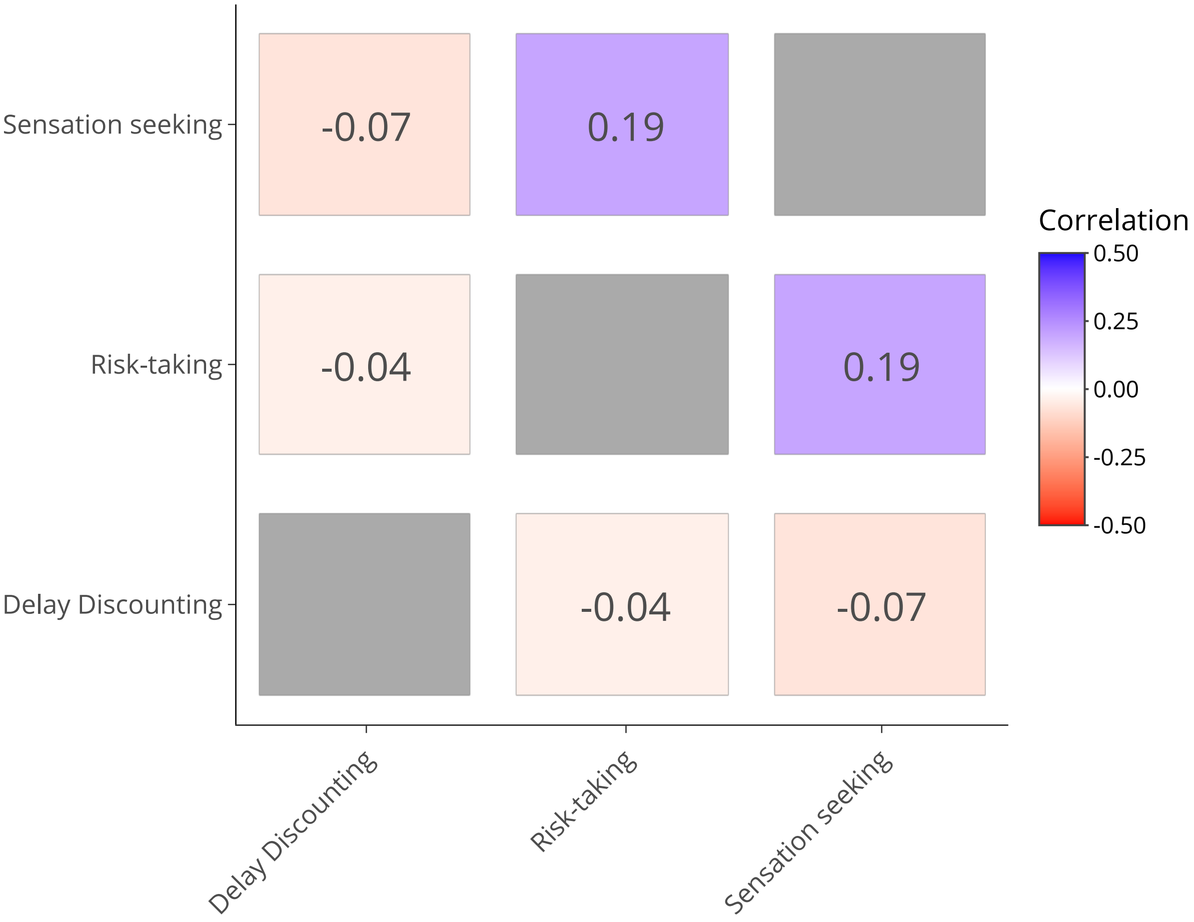

This figure shows the spearman correlations between the DRS-traits. Delay discounting seems to be slightly negatively correlated with risk-taking and sensation seeking. Risk-taking and sensation seeking share a stronger positive correlation, which is expected as the traits are often found to overlap to some extent.
